# sEMG-Based Robust Recognition of Grasping Postures with a Machine Learning Approach for Low-Cost Hand Control

**DOI:** 10.3390/s24072063

**Published:** 2024-03-23

**Authors:** Marta C. Mora, José V. García-Ortiz, Joaquín Cerdá-Boluda

**Affiliations:** 1 Department of Mechanical Engineering and Construction, Universitat Jaume I, Avda de Vicent Sos Baynat s/n, 12071 Castelló de la Plana, Spain; jortiz@uji.es; 2 Instituto de Instrumentación para Imagen Molecular (I3M), Universitat Politècnica de València, Camino de Vera s/n, 46022 Valencia, Spain; joacerbo@upv.es

**Keywords:** artificial hand, grasping postures, machine learning, EMG, recognition, HRI, low-cost devices

## Abstract

The design and control of artificial hands remains a challenge in engineering. Popular prostheses are bio-mechanically simple with restricted manipulation capabilities, as advanced devices are pricy or abandoned due to their difficult communication with the hand. For social robots, the interpretation of human intention is key for their integration in daily life. This can be achieved with machine learning (ML) algorithms, which are barely used for grasping posture recognition. This work proposes an ML approach to recognize nine hand postures, representing 90% of the activities of daily living in real time using an sEMG human–robot interface (HRI). Data from 20 subjects wearing a Myo armband (8 sEMG signals) were gathered from the NinaPro DS5 and from experimental tests with the YCB Object Set, and they were used jointly in the development of a simple multi-layer perceptron in MATLAB, with a global percentage success of 73% using only two features. GPU-based implementations were run to select the best architecture, with generalization capabilities, robustness-versus-electrode shift, low memory expense, and real-time performance. This architecture enables the implementation of grasping posture recognition in low-cost devices, aimed at the development of affordable functional prostheses and HRI for social robots.

## 1. Introduction

The design and control of artificial hands has undergone significant development in recent decades, driven by the need to enhance current devices for people with hand loss and by the growing importance of collaborative robotics and robotic grasping. Designs similar to the human hand are suitable for prosthetic use for obvious aesthetic reasons. Furthermore, they are a trend in robotics, where efforts are being made to develop adaptable end effectors that are capable of grasping a wide variety of objects, especially in assistive robotic arms like those attached to wheelchairs to support people with upper limb disabilities in performing activities of daily living (ADL) [1,2,3]. These devices increase the feeling of independence and the well-being of people with disabilities but are hardly found in commercial products due to their high associated costs and their technical requirements (battery, control, processing capacities, etc.), which constitute a drawback to their extensive use in society.

This work is aimed at improving the real-time interaction between the user and low-cost advanced artificial hands with multiple degrees of freedom (DOF), whether robotic or prosthetic. For that purpose, a natural hand control focused on the recognition and generation of ADL grasping postures is proposed, and a fast and simple machine learning (ML) algorithm able to work in low-cost devices in real time has been developed.

This paper presents the development of a simple ML technique for the real-time recognition of nine grasping postures representative of 90% of ADL from eight sEMG signals, and this is able to run efficiently (73% of success) on any low-cost platform for the development of affordable artificial hands, both prosthetic and robotic. The real-time performance and the low memory expense are key requirements for this application, as well as the independence of the technique from internet connection.

The main contributions of this paper are related to the use of ADL grasping postures for hand control:

- The use of public datasets combined with laboratory datasets in the development of the ML technique;
- The computation of only two features from the sEMG signal for fast grasping posture detection in low-cost devices;
- The robustness of the ML technique versus electrode shift and the use of GPU-based implementations.

The rest of this paper is organized as follows. Section 2 presents some previous and related work in the field. Section 3 thoroughly describes the considerations, experiments, and implementations carried out in this work. In Section 4, the results of the different experiments and algorithms are provided. An analysis of the experimental results and the final solution are given in Section 5. Finally, the paper is concluded in Section 6. In addition, three appendices expand the information provided in Section 2.

## 2. Related Work

Despite the innovations in the field of design and control of artificial hands, the use of advanced hand models is limited due to different reasons.

In the case of prostheses, the high cost of an advanced prosthesis (USD 20 k to USD 100 k) makes them unaffordable to many people and, even if they can be afforded, their lack of functionality and comfort lead to a high abandonment rate [4,5]. Instead, mechanically actuated, non-anthropomorphic one-degree-of-freedom prostheses are still extensively used. Their poor dexterous performance is due to the complex or unnatural control of prostheses with many degrees of freedom (DOF) and/or the slow response rate to user commands [4,5,6]. In fact, from the biomechanical viewpoint, hand prostheses are very simple. This simplicity is mainly due to the difficulty of establishing a proper interaction between the subject and a dexterous prosthesis with multiple DOF. This would need the existence of a human–machine interface (HMI) able to properly interpret the user’s intentions. Commercial prostheses usually present myoelectric actuation through superficial electromyography (sEMG) using the muscles of the remaining stump [7]. Other types of signals have also been used [8,9] and advances have been made, but there is a long way left to achieve simple, intelligent, and efficient prosthesis control. Although there exist advanced prosthetic hands that are mechanically capable of forming the functional grips necessary to perform ADL, the control schemes implemented in these devices are far from the coordinated control produced by the intact neuromuscular system. It should be considered that an increase in the number of degrees of freedom of the hand requires more inputs from the HMI and a high mental burden to the user. The difficulties of a fast and accurate interaction with electric hands are evidenced by the fact that the last two editions of the Cybathlon competition for arm prostheses ended with better results for simple and body-powered hands than for more sophisticated and automated models, even with osseointegration and implantable neuromuscular interfaces [10].

In the field of robotic hands, a similar tendency towards human-like designs has been observed [11]. Most of the high-tech hands have been developed by research laboratories but are rarely translated to commercial devices. Two of the most anthropomorphic developments are the Shadow Hand [12] and the DLR hand [13], where the first one has become a commercial hand, but usually offered with two or three fingers, as the whole hand is very pricy (around USD 100 k). A very sophisticated proposal for using complex sensorization and deep learning for grasping with the Shadow hand can be found in [14], and a deep and interesting survey of the problems of using ML for object recognition and grasping is given in [15]. However, the high number of DOF of these hands implies an increase in size and weight that, along with the control complexity, limit their application almost exclusively to industrial robotic arms. Even in industrial environments, manipulation is mostly performed by using simple two- or three-jaw grippers without any extra sensing [16].

Regarding human–robot interaction (HRI), as collaborative robots (cobots) are increasingly present in humans’ lives, new ways and devices for close interaction and collaboration arise. For example, during a cooperative task, robots and human partners perform complementary actions and need to exchange information to complete it. For that purpose, the robot must estimate the movement and the human intention to react efficiently and to ensure a safe collaboration. The challenge is posed by the so-called implicit communication, where information is conveyed by eye gaze, hand position and orientation, and biological signals [17]. In this sense, the sEMG signal has been used in a wide range of applications in HRI for estimating a human’s intention and movement prediction, especially in upper-limb movement kinematics.

Despite the abovementioned limitations of anthropomorphic designs, they are going to become essential in the industry and society of the near future. Regarding industry, teleoperation is now a reality in space missions and robot operation in hazardous environments; cobots that interact with humans are increasingly employed in industry; and the deployment of 5G communications and the application of artificial intelligence in the context of Industry 4.0 are going to increase the options for effective applications of more advanced, intuitive, and reliable grippers. In the field of medicine, cobots are meant to enhance the practice of medicine. Artificial intelligence, miniaturization, and the computational capabilities of computers are contributing to the increased design and use of robots in medicine [18]. Surgical interventions and other medical procedures are benefiting from the developments of artificial hands. In this sense, the higher the level of similarity between the robot manipulator and the human hand (in terms of shape and functionality), the more intuitive it will be for the medical personnel to perform a specific procedure. New social requirements are beginning to show up: the hospitality industry could benefit from the development of more skilled human-like cobots to perform service tasks, such as serving food and drinks in restaurants. Service robots are also intended to facilitate daily life for the elderly, combining the manipulation and versatility necessities to adapt to unstructured social environments [19].

Low-cost developments can make it possible to incorporate artificial hands into industrial robots, as well as make advanced prostheses accessible to people with disabilities in developing countries and also to low-income people in developed countries. Online communities such as the *Open Hand Project* [20] or *Enabling the Future* [21] have proven the power of social initiatives based on altruism and goodwill to make the world a better place for everyone.

To obtain information about the user’s intention, several sources of information have been proposed to feed pattern recognition (PR) methods. Some authors rely on machine vision, extracting information from images [22]. Others prefer to rely on dedicated sensors, such as inertial measurement units (IMU) [23]. A promising possibility is to obtain data from sEMG HMI/HRI signals [24]. Different ML approaches to solve this problem include deep learning methods (DL), fuzzy systems (FS), linear discriminant analysis (LDA), or support vector machines (SVM), among others [25]. The analysis of sEMG signals is challenging due to their stochastic nature [26]. Many confounding factors have been shown to have a great influence on the characteristics of an sEMG signal for the myoelectric control of upper-limb prostheses and, therefore, in the performance of the pattern recognition method [25]. Some of these challenges include the changing characteristics of the signal over time, electrode location shift, muscle fatigue, inter-subject variability, variations in muscle contraction intensity, as well as changes in limb position and forearm orientation [27]. To capture and describe the complexity and variability of sEMG signals for advanced applications, a massive amount of information is usually necessary.

For all of the abovementioned reasons, control, simplicity, and low-cost are very important goals in artificial hand development as they will contribute to the generalization of prosthetic hands and collaborative robotic applications in parallel with the advances of Industry 4.0.

Only a few works have tackled the detection of grasping postures for hand control. The abovementioned PR methods have mainly been used for gesture classification [28,29], requiring the computation of more than ten features from the sEMG signals and the detection of more than 15 gestures, which make them unfeasible for real-time prosthetic/robotic applications due to their high computation and memory requirements. In [30], an EMG-based learning approach is proposed for decoding the grasping intention during reaching motion. Three different PR methods were compared and no significant differences in classification performance were found. However, only three grasp types were identified, with a mean classification accuracy lower than 80%, decreasing to 60% when trying to classify five grasp types. Still, these works do not identify some of the basic grasps used by the human hand in ADL [1], which are necessary for controlling a prosthetic hand in a natural way.

Datasets are key in the development of any ML technique. To the authors’ knowledge, this is the first time that data from different datasets have been combined in the training, validation, and test of an ML technique. As indicated in [25], there is no consensus in the literature on the proper sampling rate, filtering technique, resolution, gain, etc., due to the use of different EMG acquisition devices. In this work, a Myo armband has been used, which is one of the devices used in the Ninapro Datasets, and an effort has been made to produce similar EMG data for the training, validation, and testing of the ML technique.

In addition, GPU-based implementations have been used to test many architectures and parameters with the aim of obtaining a solution that better recognizes the grasping posture intended by the user in a fast way and with the possibility of being implemented on a low-cost device, such as in Arduino or similar. In this sense, very few studies have concentrated on using either multi-threaded versions or GPU-based implementations [31].

## 3. Materials and Methods

This section is devoted to the description of the basics, experiments, and implementations developed in this work. Firstly, a description of the ADL grasping postures to be recognized from the sEMG signals gathered from the subjects is provided. Secondly, the HMI/HRI used in this work is described. It has been used to collect sEMG data from the subjects’ forearm. Thirdly, the data used in the implementation of the grasping recognition are described, along with the experiments carried out to obtain these data. Fourthly, the signal processing performed prior to the application of the recognition technique is detailed, together with the developed application. Finally, the selected technique used and the tests carried out to prove the validation of our approach are described.

### 3.1. ADL Grasping Postures

**Figure 1:**
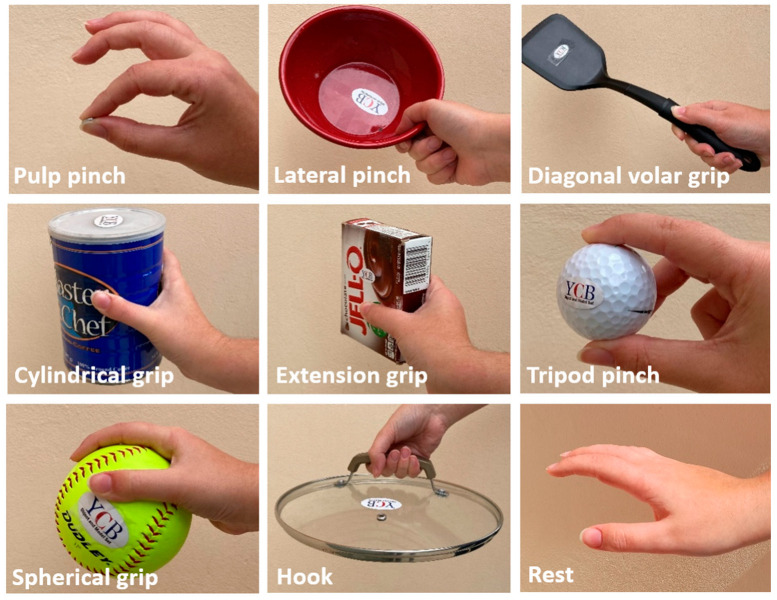
Examples of the grasp taxonomy used in this work, described in Table 1.

The grasps performed by the human hand have been classified in many ways depending on the object of the study. A commonly used classification in the field of robotic grasping is Cutkosky’s taxonomy [32], a very extensive classification developed for the mathematical modeling of the hand. Nevertheless, its high level of detail does not make it useful for the grasp classification of human grasps during ADL. The GRASP taxonomy [33] and Edwards’ classification [34] are comprehensive but also too large, with 33 and 24 different grasps considered, respectively. On the contrary, in fields such as rehabilitation [35], the taxonomies are usually poor, with differences existing among digital/whole hand or lateral/cylindrical. For that reason, our research group identified a set of grasp types in [1] that were used in more than 90% of ADL. Based on these results and previous research, an assessment protocol (AHAP [2]) was developed for anthropomorphic hands, with eight grasping postures selected for the evaluation of artificial hands. The grasps are grouped according to the interaction between the hand and the object, regardless of the number of fingers that touch the object or are involved in the grip. In the present work, these eight grasps plus the rest posture have been used, as described in Table 1. In particular and according to [2], pulp pinch and tripod pinch account for 29–48% of the grasps in ADL, lateral pinch for 9–20%, cylindrical grip for 12–25%, and the rest of grasp types for 18–36%. An example of each of these grasps can be seen in Figure 1.

### 3.2. sEMG-Based HMI/HRI

Surface electromyographic (sEMG) sensors record the electrical activity of muscles and nerves and are commonly employed to infer muscle behavior in the human body. In surface electrodes, the distance between the source and the sensor location is important and, thus, a good positioning of the electrodes plays a great role in signal acquisition. To eliminate sEMG interference and remove the low-pass filter effect resulting from the separation between the muscle and the electrode, more than one electrode is normally used, and the signal is a linear combination of them. The amplitude of the acquired signal decreases with the thickness of the tissue between the muscle and the sensor. It also depends on the force. Even though the relationship in the muscles that control the fingers is linear, other muscles have a parabolic relationship [36].

**Figure 2:**
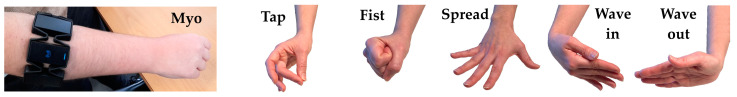
Myo armband and recognized hand postures from proprietary software Myo Connect version 1.0.1.

In this work, a commercial sEMG-based human–machine/robot machine interface has been used for data acquisition: the Myo armband from the Thalmics Lab manufacturer [37]. It is a Bluetooth 4.0 low-energy device with 8 medical grade sEMG sensors and an inertial measurement unit IMU (3-axis gyroscope, accelerometer, and magnetometer) to track forearm forces and motions, such as rotation. The sEMG sensors are evenly distributed around the entire circumference of the forearm and transform the muscle electrical signals into 8-bit integer values (ranging from −128 to 128) at a sampling rate of 200 Hz. Once correctly located on the forearm, calibrated, and trained, it can recognize 6 hand gestures with the manufacturer software: tap, fist, open, wave in, wave out. These gestures are shown in Figure 2.

### 3.3. Datasets

In this study, two types of datasets have been used in the development of the deep learning technique: data for training and data for validation. The datasets proceed from intact subjects from the well-known NinaPro Database [38,39] and from experiments in our laboratory. Both datasets are detailed in the following subsections. According to [40], healthy subjects can be used as a proxy measure for transradial amputees, although with a slightly lower performance (around 15% less).

Other datasets have been considered, such as the Myo Dataset in [28], but they do not include all the ADL grasping postures, which would lead to an unbalanced result for the DL technique.

#### 3.3.1. NinaPro Dataset 5

sEMG data from the NinaPro Dataset 5 (DS5) database [39] have been used for training purposes. NinaPro [38] is a public database that stores data from hand motions with different EMG sensors to help in the research of human grasping and in the realization of robotic or prosthetic hands. The database is made up of 10 different datasets that differ in the technology used to collect the data and the number of experimental subjects.

DS5 provides data from 10 right-handed healthy subjects, 8 males and 2 females, performing 53 hand postures using two Myo armbands placed on the largest diameter part of the forearm and aligned together, one upper and one lower. The upper Myo is close to the elbow (typical configuration for this device). The lower Myo is located just below (closer to the hand) and is rotated 22.5° with respect to the previous one. According to the dataset developers, this setup was used because it leads to excellent mapping of the forearm muscles [39].

In the dataset, a total of 53 different movements are performed with 6 repetitions, each lasting 5 s for every one of them. There is a 3 s resting time between movements in which the subject’s hand lies in a rest position to avoid muscle fatigue. The movements are organized in 3 types of exercises: A, B, and C. In each exercise, the subject performs all the positions in a row (separated by the six repetitions and by the 3 s rest time). The different movements composing the dataset can be seen in [38], and those belonging to exercise C can be classified into the grasp types described in Section 3.1. Exercises A, B, and D do not include object grasping tasks, but motions of single fingers or the whole hand.

**Figure 3:**
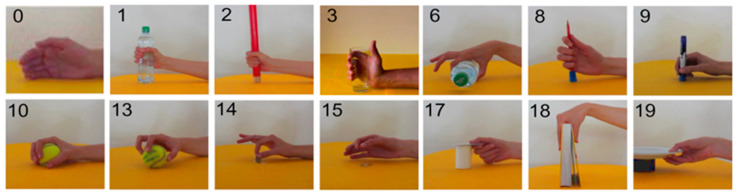
Grasping postures from Ninapro DS5 (with ID). Figures extracted from [38].

The data for training purposes have been selected from DS5, Exercise C. In Table 2 and Figure 3**,** the selected grasping postures are shown, along with their identification number (ID) in NinaPro DS5 and the grasped object. The rest posture, identified by the number 0, can be found in all the exercises.

#### 3.3.2. Experimental Data

Training and validation data have been collected from laboratory grasping experiments (UJIdb) specifically designed for the purpose of this work. They were performed using the using the Myo armband from Thalmics Lab, Kitchener, ON, Canada. In addition, due to data protection considerations, the subjects’ data were encrypted and stored in the database of the Biomechanics and Ergonomics Group.

The subjects participating in the study had the following characteristics:

- 10 healthy subjects
  ○ 5 male, 5 female.
  ○ 9 right-handed, 1 left-handed.
- The age ranged from 18 to 60 years old.

The objects involved in the grasping experiments were selected from the Yale-CMU-Berkeley (YCB) set of objects [41,42,43]. The YCB Object and Model Set is a set of objects, models, and protocols that owes its name to the universities that designed it: Yale University, Carnegie Mellon University, and the University of California, Berkeley. It is designed for the evaluation of dexterous hands in robotics and consists of a set of everyday objects with different sizes, shapes, weight, texture, and rigidity. The set has a total of 77 objects separated into different categories based on their utility: food, kitchen, tools, shapes, and tasks [44]. The function of this set of objects is to facilitate benchmarking so that all the researchers carry out their experiments with the same objects [41,42].

**Figure 4:**
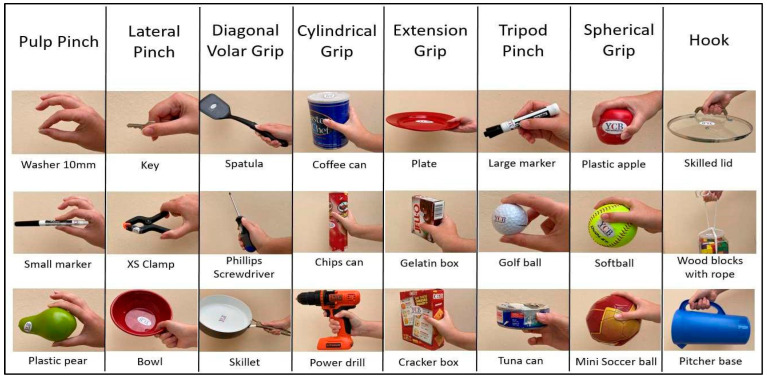
Objects from the YCB set used in the grasping experiments associated with a grasp type.

In this study, some of the objects from the YCB set were selected by considering the most suitable grasp for each object, as in [2]. For each grasp type in Table 1 (except for the rest posture), three different objects were selected to perform the grasping experiments. Figure 4 depicts the selected objects and the associated grasp type.

A data collection application was developed to acquire data from the 10 subjects performing the 8 grasps proposed, with the YCB set objects (Figure 4) plus the rest posture (with no object involved). Therefore, a total of 25 different hand postures were performed. This application was designed using the App Designer tool from MATLAB [45].

**Figure 5:**
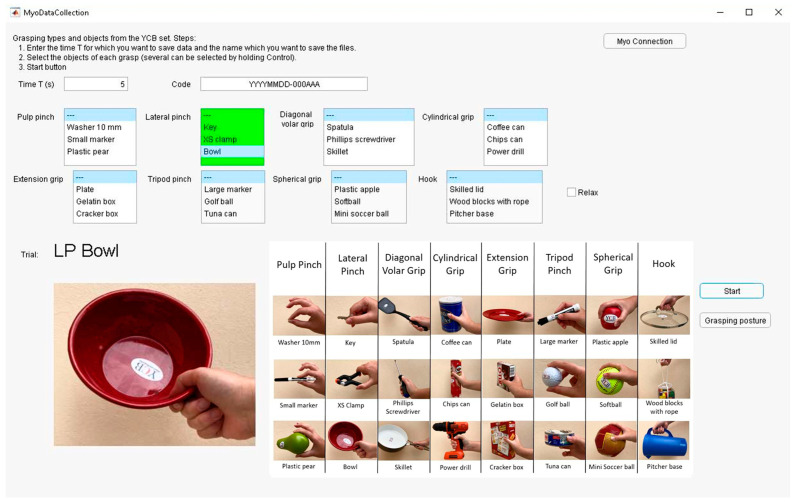
Snapshot of the data collection application.

In the application, the user can select the grasps to be performed and the objects to be grasped in each trial, as shown in Figure 5. In the lower left corner of the screen, the text and image change depending on the object and grasp to be performed. This visual output facilitates automatic data collection with little intervention from the operator.

The data acquisition protocol that was followed by every subject is described in Appendix A. These experiments were carried out with the 10 subjects, and each subject performed the grasping movement 3 times for each object. The data collection application stored the data from the 8 sEMG and the IMU sensors in MATLAB variables (.mat), which were exported to CSV files once the session was finished.

### 3.4. Data Preprocessing: Feature Extraction

The raw datasets gathered for training and validation purposes were not directly used in the DL technique. They were previously processed with the aim of obtaining the key features of the sEMG signals.

Feature extraction is a method to obtain the useful hidden information in sEMG signals and remove interferences, as indicated in [46]. For the classification to succeed, the selection of the feature vector is of capital importance and redundant features must be avoided. In [46], the properties of thirty-seven time and frequency domains features were studied. The results indicated that frequency-domain features were not suitable for EMG recognition systems and that most time-domain features were superfluous and redundant. Time-domain features can be grouped into four main types: energy and complexity, frequency, prediction model, and time dependence.

The findings in study [46] regarding these types of features were:

- Prediction model features have poor ability in classification tasks.
- Time-dependence features do not outperform features in the energy and complexity group.
- When the number of features is greater than 2, the classification accuracy only has a slight increase.

Attending to the recommendations given in [46] regarding the selection of features for EMG signal classification and the results in [28], the following ten descriptors were used in this study, all of them belonging to the time-domain class [46]: Mean Absolute Value (*MAV*), Integrated EMG (*IEMG*), Root Mean Square (*RMS*), Waveform Length (*WL*), Zero Crossing (*ZC*), Slope Sign Changes (*SSC*s), Skewness (Sk), Activity (Ac), Mobility (Mob), and Complexity (Cx). The descriptions and equations of these features can be found in Appendix B. It is worth noting that our selection includes the successful Hudgins’s EMG feature set [47] for testing (*MAV*, *WL*, *ZC*, *SSC*s).

One of the goals of this work was to develop an efficient grasping recognition technique able to work in low-cost devices in real time. With this purpose in mind and taking into account the previous considerations, the objective was to obtain the two features that best recognize the 9 grasping postures detailed above. Thus, the selected AI technique for grasping recognition was trained with all the possible combinations of the 4 features to check whether the difference in recognition rate was as small as stated in [46] and whether it could outperform Hudgin’s feature set.

### 3.5. Pattern Recognition (PR) Methods

Many artificial intelligence and machine learning techniques are used to recognize patterns in EMG signals [48]. Among them, the most used in arm muscle activity recognition are [24,25]: deep learning (DL) methods, fuzzy logic (FL), linear discriminant analysis (LDA), the k-Nearest Neighbors (KNNs) algorithm, decision trees (DTs), and support vector machines (SVMs). The main characteristics are described below to justify the selection of the technique used in this work.

#### 3.5.1. Description of PR Techniques

DL methods are those based on neural networks (NNs) of different configurations and architectures. These methods are widely used to predict any type of pattern and to spot trends because of their robustness against variability in signals that, like EMG signals, are uncertain and non-linear. In addition, they are good at generalization once trained, so that new data can be introduced and satisfactory results can be obtained. A variety of architectures have arisen with different properties: the radial basis networks, for instance, are commonly used, but they require more neurons in the hidden layer than the typical multi-layer perceptron (MLP), increasing the number of hits to be used. Recent types of neural networks widely used for their great effectiveness are convolutional neural networks (CNNs) and recurrent neural networks (RNNs). The main drawback of these new networks is that they tend to be computationally expensive and thus ill-suited for embedded systems [28], such as those required when guiding a prosthetic. In addition, the way these networks work is so unpredictable that sometimes the result obtained cannot be understood.

Linear discriminant analysis consists of finding the optimal direction to which data are directed. The main advantages of this technique are efficiency and simplicity, although the computation time is long. This technique is normally used in conjunction with principal component analysis (PCA), which allows for the dimensions of the original data to be reduced. The technique takes the original data and calculates some characteristics that can simplify its dimensions, with a minimum loss of information.

The k-Nearest Neighbors algorithm is very popular due to its simplicity and good results. This algorithm groups data into so-called “regions”. When new data enter the system, the algorithm defines which region they belong to. As downsides, this technique is sensitive to irrelevant attributes of the data, to outliers, and to missing data, and it is computationally expensive at testing as it needs to store all the training examples and compute distances to all the examples.

Decision trees are an artificial intelligence technique that progressively classify data into small stages, where each stage depends on the previous one. One of the advantages of this method is that is very easy to understand. A variation of this method is random decision trees (RDTs), where several trees are trained with part of the input data (packet) randomly separated from each other. Many trees (one for each packet) are created, forming a random forest. Data to be classified are introduced in all the trees and a voting system provides the result, with the most repeated value in the entire forest. The main disadvantage of this method is that if there are many features, the trees become very large and take a long time to execute.

SVM consists of generating an optimal hyperplane in a multidimensional space to separate different classes. The name of the technique derives from one of its components, the support vectors, which are the closest data points to the hyperplane of each class. The perpendicular distance between support vectors is called the *margin*. The optimal hyperplane is the one with the largest margin. This type of AI has good accuracy and is faster than other algorithms, but it only works if there is a clear margin of separation. Also, it is not suitable for very large datasets or if the sets overlap.

#### 3.5.2. Selection of the PR Technique

The goal of the present work was to develop a PR technique able to identify 9 grasping postures from 8 sEMG signals in real time on a low-cost platform such as Arduino, NodeMCU, Teensy, etc. The *real-time performance* and the *low memory expense* were requirements with a great influence on the selection of the technique to be used. In fact, methods such as LDA, DT, and kNN were discarded due to the real-time requirement. Similarly, complex DL networks (CNN, RNN, etc.) and DT were discarded due to memory requirements and, finally, the SVM method was not suitable for sets that overlap, which is the case.

**Figure 6:**
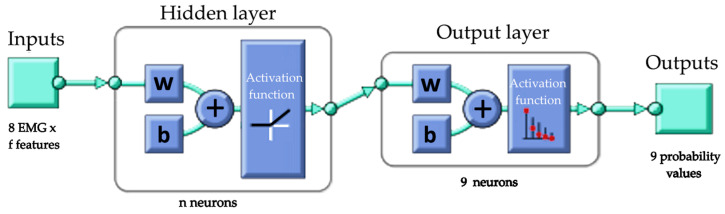
Architecture of the multi-layer perceptron proposed for grasping posture recognition.

Therefore, a simple MLP was selected with one hidden layer. The general architecture of the NN is shown in Figure 6, where some of the parameters will be selected from the tests explained in the next section (number of features, number of neurons in the hidden layer, and training and activation functions). This architecture has been proven to be valid for addressing similar problems in previous authors’ work [49]. Its main advantages are:

- Robustness against uncertainty in the signal;
- Generalization capabilities;
- Low memory expense (with low number of neurons in the hidden layer);
- Real-time performance of the trained model.

The selection of such a simple architecture could have the disadvantage of a longer training time to converge to the optimum state than new PR techniques due to the large EMG dataset. According to [25], research based on big EMG datasets can be tackled by either modifying traditional methods to run in parallel computing environments or by proposing new methods that natively leverage parallel computing. In this work, the first option has been chosen; that is, the MLP method has been modified to run in parallel to overcome the long training time.

#### 3.5.3. A Machine Learning Approach for Grasping Posture Recognition

In this section, the design of the MLP architecture used for grasping posture recognition is described. The implementation was carried out with the MATLAB Deep Learning Toolbox.

The architecture to be tested was a fully connected 2-layer feedforward NN with a hidden layer implemented in MATLAB with the *patternnet* function. Many tests were performed to obtain the optimal NN capable of recognizing the different selected grasping postures, avoiding underfitting and overfitting. Some of the hyperparameters of the NN were repeatedly changed to test the performance of the network according to [50]. In particular, the following tests were carried out:

- *Test 1: Input vector: datasets and features*
  Input data from the DS5 and the UJIdb were used for feature computation (10 features described in Section 3.4) and later used in the training and testing phases, thus obtaining a considerable amount of data. Globally, a total of 380,000 descriptors were calculated to train the network and 40,000 descriptor data were saved only for testing.
  The tests were run with the calculated features as input data from the different databases separately and jointly to identify the best results. In each test, the data from one subject were excluded from training and used in testing. All 385 combinations of 1, 2, 3, and 4 features were tested as input data.
- *Test 2: Number of neurons of the hidden layer.*
  Based on the results obtained in [49], the tests started with 100 neurons in the hidden layer, with this number being increased or decreased in amounts of 25 or 50 ranging from 50 to 300: {50, 75, 100, 125, 150, 200, 250, 300}.
- *Test 3: Activation function of the hidden layer*
  Tests were performed with the following activation functions, described in Appendix C: {*elliotsig*, *hardlim*, *hardlims*, *logsig*, *poslin*, *purelin*, *radbas*, *satlin*, *satlins*, *tansig*}
- *Test 4: Training function*
  Tests were performed with the following training functions, described in Appendix C: {*trainbfg*, *traincgb*, *traincgf*, *traincgp*, *traingd*, *traingda*, *traingdm*, *traingdx*, *trainoss*, *trainrp*, *trainscg*}
- *Test 5: Weights of neurons in the hidden layer*
  Tests were made with and without their initialization to the best solution to assess the NN performance.
  Also, the following additional tests were performed:
- *Test 6. Data window and overlapping sizes*
  The sliding window approach was used as it allowed us to increase the size of the training set (data augmentation) and it induces robustness to noise in the learned model, which leads to a better generalization.
  Different sizes of the pair {*data window*, *data overlap*} were tested, including those recommended in the literature [28,39]: {20, 10}, {200, 100}, {52, 47}.
- *Test 7. Robustness against HRI displacement*
  Different groups of input data from the DS5 and the UJIdb were tested, which implied the use of different HRIs: {*DS5 upper Myo*, *DS5 lower Myo*, *DS5 upper Myo* + *UJIdb*, *DS5 lower Myo* + *UJIdb, DS5 upper Myo* + *DS5 lower Myo* + *UJIdb*}. In each one of these databases, data from one subject were excluded from training and used in testing. The 10 features described in Section 3.4 were computed for all these data. All 385 combinations of 1, 2, 3, and 4 features were tested as input data.
- *Test 8. Multiple neural networks*
  The efficacy of using three smaller NNs instead of one to identify different grasping postures and choose the correct one using a voting system was tested.
- *Test 9. Parallelization*
  Tests were made using the parallelization provided by MATLAB. This parallelization could imply the use of the CPU nodes and/or the use of the GPU nodes in parallel. Not all the functions provided by the Deep Learning Toolbox could be used by the GPU nodes.
  The results of these tests were assessed according to the following variables:
- *MSE* (Mean Squared Error) among the NN outputs ($x_{i}$) and the desired values (targets, $t_{i}$) for each EMG sensor and each posture. This value was calculated for each NN with the subjects’ data specifically separated for the test phase and it was slightly higher than the one obtained in MATLAB, which was obtained from randomly isolating data from the different subjects used for training.
  (1) $$MSE=\frac{1}{L}\sum_{i=0}^{L-1}{\left(x_{i}-t_{i}\right)}^{2}$$
- *RMSE* (Root Mean Square Error) among the NN outputs and the targets for each EMG sensor and each posture, calculated as the square root of the *MSE* value.
  (2) $$RMSE=\sqrt{\frac{1}{L}\sum_{i=0}^{L-1}{\left(x_{i}-t_{i}\right)}^{2}}=\sqrt{MSE}$$
- *Error percentage*, which is the percentage of times in which the output and the target are not the same for a given sample. For this purpose, the posture with more probability was considered the selected one.
- *Performance* value, which is the value of the performance function of the NN that can be selected among those detailed in Appendix C, Table A3. It was set to *MSE*. The final value of the performance function will not be the same as the average *MSE*, since the performance value is computed using the test data automatically selected by the NN, while the actual *MSE* is calculated with a test subject not entered in the network. The main difference is that the NN saves random data for testing purposes and not only from one subject, as is desired to evaluate the prediction capabilities. Therefore, the *performance* value will, in general, be smaller than the *MSE* value due to this difference.
- *Confusion matrix*, which indicates the times an output is equal or different from the target for each posture. Diagonal values indicate the number of successful outcomes (output = target), while off-diagonal values indicate misassignments (output ≠ target). The best confusion matrix would be a diagonal matrix, as each posture would be recognized appropriately. This matrix allows us to identify which grasps have similar EMG signals.

## 4. Results

In this section, the results of the different tests detailed in Section 3.5.3 are described. These tests have been carried out with the methods explained above and have led to a specific NN architecture, a two-layer feed-forward neural network, which provided the best results in terms of the lower global *RMSE* for all the postures.

In the following paragraphs, some insights are given into each test instead of the specific data, this is for the sake of clarity and brevity due to the high number of tests carried out. Later, the proposed architecture is detailed.

- *Test Results 1: Input vector: datasets and features*
  The better *RMSE* results of the different NNs ranged from 26.96% to 27.46% for combinations of the features *MAV*, Skewness, activity, complexity and *IEMG*. The rest of the combinations produced higher *RMSE* results (reaching 38.65%).
- *Test Results 2: Number of neurons of the hidden layer.*
  With the progressive increase in the number of neurons in the hidden layer, a progressive increase in the mean *RMSE* was observed. In fact, a minimum mean *RMSE* value was identified for around 100 neurons in the different tests, which is consistent with previous experimentation [49]. Therefore, this number of neurons is generally adequate for the type of architecture and problem proposed.
- *Test Results 3: Activation function of the hidden layer*
  The activation functions *hardlim*, *hardlims,* and *logsig* provided very high average *RMSE* values, implying high error in the outcomes. Furthermore, it was observed that the *purelin* activation function could not achieve many results due to the shape of the function. This is consistent with the fact that the rectified linear transfer function and enhanced versions are recommended for multi-layer perceptron [51].
- *Test Results 4: Training function*
  The training functions *traingd*, *traingda*, *traingdm*, *traingdx,* and *trainrp* are not adequate for the proposed architecture due to the very high *RMSE* values of performance in all cases.
- *Test Results 5: Weights of neurons in the hidden layer*
  This test provided similar *RMSE* values for networks with and without the weights of the hidden layer initialized with the best solution.
- *Test Results 6. Data window and overlapping sizes*
  The features were calculated in a window of 52 data (260 ms) with an overlap of 47 data (235 ms), as in [28]. The pair {52, 47} of {*data window*, *data overlap*} produced the best *RMSE* values in general. This size of overlap is small enough (5 values) to maintain the characteristics of the signal in the recalculation.
- *Test Results 7. Robustness against HRI displacement*
  Similar results have been obtained with all the configurations, with the lower Myo slightly better than with the original configuration. Therefore, the algorithm is robust against variations in the position and orientation of the collection device since the different grips can also be recognized with similar *RMSE* value (26–27%).
- *Test Results 8. Multiple neural networks*
  The use of multiple NNs provided higher *RMSE* values (around 38%) than the initial architecture. Therefore, the use of several NNs for grasping classification is not recommended.
- *Test Results 9. Parallelization*
  The use of parallelization on the GPU showed some difficulties: the need to relocate the data in a specific *gpuArray* data type with less precision than the original data, and the fact that some activation and training functions cannot be used on the GPU. Still, the parallelized training in both devices, the CPU and the GPU, was mandatory, since it made it possible to perform the tests described in this work.

The architectures that obtained the best results from all the tests performed are described in Table 3. The least global *RMSE* for all the postures was obtained with configuration 1 in Table 3 which, in addition, only used two features: *MAV* and Skewness. For that reason, this is the one selected for the recognition of the nine grasping postures. Configurations 2, 3, and 4 needed three or four features, which implies additional computational time and no better performance.

**Figure 7:**
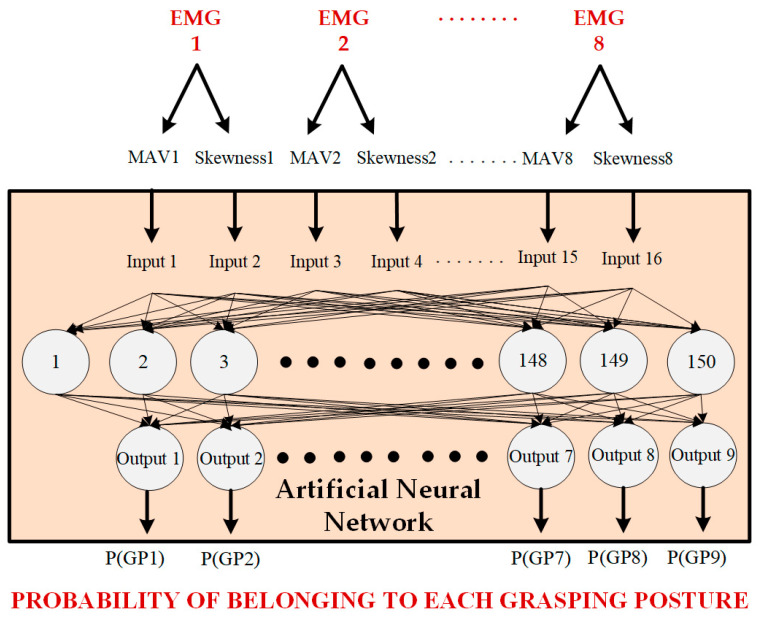
MLP for the recognition of 9 grasping postures with two features (*MAV* and Sk).

In the selected network, a window of 52 data and an overlap of 47 data were used for feature calculation from the EMG data, as described in [28]. The network only has one hidden layer with 150 neurons, which produces a simple architecture, as can be seen in Figure 7. The NN was trained using conjugate gradient backpropagation training with Polak–Ribiére updates for the computation of the neuron weights according to the formulation in [52,53,54]. The hidden layer activation function is a linear and positive activation function, consisting of a zero output for negative data and a linear output for positive data. Finally, the activation function of the output layer allows the outcomes to be the probability values of the different grasping postures.

The performance function selected to minimize towards the goal is the *MSE*. The choice of this parameter was due to the fact that the *RMSE* can be calculated from it. Therefore, minimizing the *MSE* implies minimizing the *RMSE*. Thus, for every computed NN, the mean *RMSE* of all the grasping postures has been calculated to check the efficiency of the network against the validation data. Table 4 shows the *MSE* and *RMSE* values for the adopted solution described in this section: the global values as well as those for each hand posture. Note that the global *MSE* parameter computed by MATLAB is 0.0758, which is slightly lower than the one calculated with the subjects separated for the test phase, as shown in Table 4.

**Figure 8:**
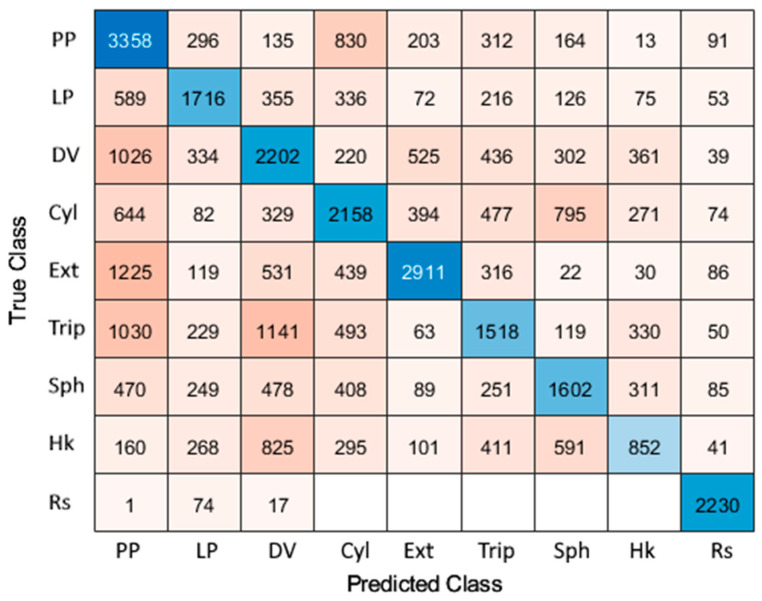
Confusion matrix for the solution neural network with a subject not used for training.

The confusion matrix associated with the classification results for a subject not used for training is depicted in Figure 8 in a colored manner. Here, diagonal cells appear darker the better the classification results they show (output = target) are, and off-diagonal cells appear clearer the less misassignments (output ≠ target) they show. This matrix gives clues about the postures that are being mixed and in what sense the classification can be improved.

Finally, to check the viability of a low-cost implementation of this solution, the time needed to carry out all the necessary operations was calculated. The acquisition time of the 52 data required to compute the descriptors by the Myo device is around 260 ms. The calculation time of the two descriptors of the solution is 3.23 ms and it takes 337.43 ms to go through the network without using the different cores of the computer or the GPU. Globally, the time invested in recognizing a grasping posture once the muscle contraction has been performed is 600.66 ms from when the instant data collection begins. This time has been computed in MATLAB and it would be shorter on a low-cost microprocessor. The amount of time required is quite small, so grasping posture recognition seems feasible for any low-cost application with the proposed algorithm.

## 5. Discussion

The objective of this work was to implement a simple deep learning approach able to recognize nine daily-life hand postures in real time with the best possible performance for it to work on a low-cost robotic/prosthetic hand. Other applications could benefit from this approach, such as the teleoperation of a robot hand in dangerous applications or the rehabilitation of forearm muscles in amputees through connection with a virtual reality hand.

The final NN architecture selected (ID 1) is described in Table 3 and Figure 7. It is a feed-forward neural network with a 150-neuron hidden layer, which constitutes a very simple type of network in terms of memory and computational requirements. The network has 16 inputs and 9 outputs. The inputs are the two descriptors, *MAV* and Skewness, obtained from each EMG signal of the Myo armband (eight EMG sensors). The outputs are the nine hand postures. In this network, a window of 52 data (260 ms) and an overlap of 47 data have been used for feature calculation, as described in [28]. A maximum latency of 300 ms is recommended in [47]. This type of overlap allows the amount of new data introduced in the recalculation of the descriptor to be very small.

The *RMSE* values shown in Table 4 confirm that the network presents good efficiency, since it is capable of recognizing the different grasps of different subjects with a small error for subjects different than those used for training, and it is even lower for the same subject. In particular, a global *RMSE* of 26.96% was obtained, which implies a rate of success of 73.04%. It is worth noting that the error is not the same for all grasp types. For the pulp pinch, diagonal volar grip, cylindrical grip, and tripod pinch, an efficiency of slightly less than 70% was obtained. The lateral pinch is one of the best developed, with an efficiency of 75.3%, but above all, the one that best differs is rest, with 90% success. This is due to the fact that rest is not really a grasping posture and the muscular activity is almost non-existent, differentiating it greatly from the other grasps. Even so, the difference in performance between grasps is small (there are no grasps with 80% efficiency and others with 20%), exhibiting a good characteristic of the network. The fact that they all have a similar performance adds value to the algorithm, since there is no grasp that is always misassigned.

However, the efficiency of the NN algorithm could be enhanced. Further work on this matter will deepen. In this sense, the confusion matrix indicates the postures that are more likely to be confused. For instance, the DV, Ext, and Trip are likely to be confused with PP. On the other hand, PP is usually correctly classified, although sometimes it is confused with Cyl. Similarly, Ext and Hk can be confused with DV, and Cyl can be confused with Sph. These misassignments can be attributed to the fact that some of the grasping postures are quite similar. To distinguish the grasping postures properly, we could add analogous but smaller networks to specifically deal with predicted PP and DV postures. This way, a two-step NN algorithm could be developed that would increase the success rate.

In addition, the real implementation of this technique will depend on the hand to be controlled. Most of the artificial hands that are currently on the market do not have all the DOF of the human hand, with 5 or 6 being the maximum number of DOF in general. In such cases, some of the postures could be merged. For instance, fingers 3 to 5 are usually moved with one flexion–extension motor. Therefore, tripod grasping posture cannot be generated. In addition, artificial hands often lack abduction–adduction movements of the fingers. At most, the thumb is the only finger with this type of motion. Therefore, spherical and cylindrical grasps will be identically performed, and it makes no sense to distinguish them. All these concerns will be addressed in the future.

## 6. Conclusions and Further Work

The work described in this paper had the goals of successfully identifying the grasping postures most used in daily life with good efficiency through a sEMG Myo armband, as well as identifying a simple machine learning approach that can be be implemented on low-cost platforms, such as Arduino or Teensy. This was is based on a feed-forward neural network with a hidden layer for pattern recognition. More complex DL algorithms could not be used in low-cost devices due to their high memory and computational requirements.

For the development of this work, electromyographic data from the forearm of 10 healthy subjects were collected in a completely satisfactory manner. The data acquisition device, together with the application designed and used in the experimental tests, can be used in subsequent work to collect more information from subjects with the aim of enhancing efficiency.

The data obtained during the trials were used in conjunction with data from the NinaPro DB5 external database for training and validation purposes. In addition, we verified that the developed algorithm deals with uncertainty well due to the unwilling displacement of the acquisition device. This makes the obtained solution robust against changes, both in the subject and in sensor location.

As an artificial intelligence technique, neural networks have been found to be a good technique for recognizing human grasp. In addition, good efficiency has been achieved with a simple architecture consisting of a fully connected single-layer feedforward backpropagation neural network. This network does not fall into misalignment or overadjustment problems, since it provides good results with new data, recognizing 73.04% of the grasping postures made by the user. This means that it can be implemented in low-cost microprocessors, or even that efficiencies can be improved by increasing the number of hidden layers and using other types of more complex architectures.

In summary, this work will allow us to recognize hand grasps in all kinds of applications, whether in the prostheses being built at the Biomechanics and Ergonomics Group of the Universitat Jaume I or in artificial hands for the industrial field.

Future work in the prosthetic field implies testing the technique on subjects with transradial amputation implementation, and also assessing the data collection and ML techniques through an EMG-based Myo armband using a low-cost microprocessor in a low-cost artificial hand. This microprocessor must be able to collect data from the Myo in real time, calculate two features, run through the ML algorithm, and recognize the hand posture that is being performed.

Further work in robot hands would involve the use of this technique in the teleoperation of a low-cost hand, such as the RH8D hand available at our lab. The teleoperation of an artificial hand would allow for tasks such as remote surgical intervention or operation in hazardous environments.

## Figures and Tables

**Table 1:** Description of the grasps used in this work.

ID	Grasp Name	Description
PP	Pulp Pinch	Only the thumb and the tip of the fingers are used. Unused fingers may be in extension or flexion.
LP	Lateral Pinch	The lateral part of the fingers (one or more) is used, and usually the thumb too. The rest of the fingers are flexed.
DV	Diagonal Volar Grip	Variant of the cylindrical grip when the object is not parallel to the palm. In this case, the thumb is abducted, parallel to the object.
Cyl	Cylindrical Grip	The palm is involved during the grasp as it touches the object and is arched. The thumb is in direct opposition to the rest of the fingers.
Ext	Extension Grip	The thumb and proximal part of the fingers are involved in the grasp, but the palm is not.
Trip	Tripod Pinch	The thumb and two more fingers are used, being able to use the tip or the side of the latter.
Sph	Spherical Grip	The hand curves to hold the object with all the fingers abducted and with the intervention of the palm.
Hk	Hook	The palm and the thumb are not involved in the grip since the entire weight of the object is held by the fingers.
Rs	Rest	The fingers and the palm are not exerting any force.

**Table 2:** Training data used from Dataset 5 in the Ninapro Database.

Grasp Type	ID in Ninapro DS5, Exercise C	Objects
Pulp Pinch	6	Small Water Bottle
	15	Coin
Lateral Pinch	14	Coin
Diagonal Volar Grip	8	Pencil
	17	Card
Cylindrical Grip	1	Large Bottle
	2	Handle
Extension grip	18	Book
	19	Plate
Tripod pinch	9	Marker
	13	Tennis Ball
Spherical grip	10	Tennis Ball
Hook	3	Tall Glass
Rest	0	None

**Table 3:** Parameters of the networks with best *RMSE* performances (see functions in Table A3).

Neural Network ID	1	2	3	4
**Type of architecture**	Feed-forward neural network with a hidden layer			
**Number of features as inputs**	2 × 8 EMG 16 input arrays	3 × 8 EMG 24 input arrays	3 × 8 EMG 24 input arrays	4 × 8 EMG 32 input arrays
**Descriptors/features**	*MAV* & Sk	*MAV* & Ac & Sk	Cx & *IEMG* & Sk	Ac & Cx & *IEMG* & Sk
**Mean *RMSE***	0.2696	0.2706	0.2717	0.2706
**Training function**	traincgp	traincgb	trainoss	trainscg
**Neurons in the hidden layer**	150	120	150	75
**Activation func. (hidden layer)**	poslin	satlins	elliotsig	radbas
**Activation func. (output layer)**	softmax	softmax	softmax	softmax
**Performance function**	mse	mse	mse	mse

**Table 4:** *MSE* and *RMSE* results for the grasp types in the solution neural network.

Grasp Type	*MSE*	*RMSE*
Pulp pinch	0.0941	0.3068
Lateral pinch	0.0610	0.2470
Diagonal volar grip	0.1042	0.3229
Cylindrical grip	0.0987	0.3141
Extension grip	0.0794	0.2818
Tripod pinch	0.0958	0.3096
Spherical grip	0.0754	0.2747
Hook	0.0716	0.2676
Rest	0.0105	0.1022
Total	0.0768 ± 0.0287	0.2696 ± 0.0675

**Figure A1:**
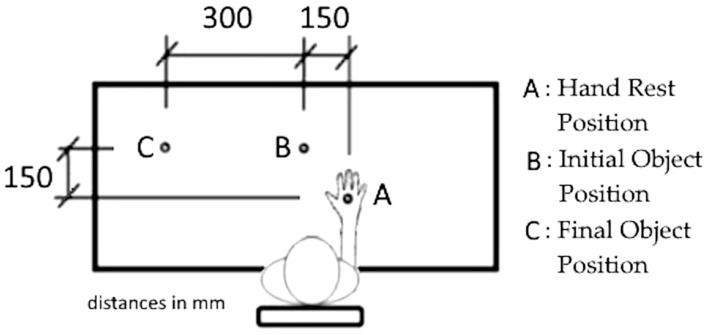
Setup for the grasping experiments: table with the hand rest position (A) and initial (B) and final (C) object positions for grasping.

## Data Availability

The data presented in this study from the NinaPro Database are openly available at https://ninapro.hevs.ch/instructions/DB5.html (accessed on 21 March 2024). The data from UJIdb presented in this study are available upon request from the corresponding author.

## Appendix A. Data Acquisition Protocol

The data acquisition protocol followed by every subject was composed of several steps:

1. The subject sits on a table and the operator places the Myo armband on the right subject’s forearm.
2. The subject synchronizes the Myo armband with the computer, following the operator’s instructions.
3. The data collection parameters are introduced by the operator: time (5 s), subject’s ID, and grasps to be performed. The operator thoroughly explains to the subject how to carry out each grasp.
4. The operator selects the grasp, locates the object in the initial position (B), presses the “Start” button, and waits for the image of the selected grasp to appear in the left-bottom corner of the data collection application (Figure 5).
5. Once the grasp image appears, the user initiates the pre-grasping motion with the right hand from the rest position (A) on the table to the initial object position (B), lifts the object, stays still until the image disappears (in the meantime, the operator captures the “Grasping posture” by pressing the corresponding button), and releases the object in the final object position (C). These positions are shown in Figure A1.
6. The subject returns the hand to the rest position on the table.
7. Steps 4, 5, and 6 are repeated 3 times.
8. To continue the experiments, go to step 4. Otherwise, continue to step 9.
9. The operator removes the Myo armband from the subject.

## Appendix B. Selected Features

The equations are considered on a data window of length L, where the *k*-th element of the *i*-th window corresponds to *x_i,k_*.

1. Mean Absolute Value (*MAV*). It is the mean of the absolute value of the sEMG signal (fully rectified signal). This descriptor is commonly used for EMG-based monitoring applications as it can detect muscle contraction levels.
  (A1) $$MAV(x_{i})=\frac{1}{L}\sum_{k=0}^{L-1}\left|x_{i,k}\right|$$
2. Integrated EMG (*IEMG*). It is the sum of the fully rectified signal, and it is related to the EMG signal sequence firing point [46].
  (A2) $$IEMG(x_{i})=\sum_{k=0}^{L-1}\left|x_{i,k}\right|$$
3. Root Mean Square (*RMS*). This parameter is closely related to the standard deviation, since both are equal when the mean of the signal is zero. It is calculated with the following equation:
  (A3) $$RMS(x_{i})=\sqrt{\frac{1}{L}\sum_{k=0}^{L-1}x_{i,k}^{2}}$$
4. Waveform Length (*WL*). It is a simple characterization of the waveform, since it is the accumulated length of the waveform in a given time.
  (A4) $$WL\left(x_{i}\right)=\sum_{k=1}^{L-1}\left|x_{i,k}-x_{i,k-1}\right|$$
5. Zero Crossing (*ZC*): It reflects the frequency at which the signal passes through zero, that is, the number of times the signal crosses the y-axis. The values of the signal collected through the Myo armband provide values between −128 and 128, centered in zero.
  (A5) $$ZC(x_{i})=\sum_{k=1}^{L-1}\left(\left(\left(x_{i,k}-x_{i,k-1}\right)\geq 0\right)\wedge \left(sgn(x_{i,k}\right)\neq sgn\left(x_{i,k-1}\right)\right)$$
  where ϵ is a threshold parameter. Boolean operations such as ≥ or ≠ evaluate to 1 if TRUE and to 0 when FALSE.
6. Slope Sign Changes (*SSC*): It measures how often the sign of the signal slope changes. The number of changes between a positive or a negative slope in three consecutive samples is computed, where a threshold parameter (ε) is included to avoid the noise in the sEMG signal.
  (A6) $$SSC\left(x_{i}\right)=\sum_{k=1}^{L-1}f\left[\left(x_{i,k}-x_{i,k-1}\right)\cdot \left(x_{i,k}-x_{i,k+1}\right)\right],\;with\;f\left(x\right)=\left\{\begin{array}{c} 1,\;\;\;if\;x\geq \epsilon \\ 0,\;otherwise \end{array}\right.$$
7. Skewness (Sk): This parameter is the third central moment of a distribution and measures the asymmetry of a distribution. It is computed using Equation (A5), where $\bar{x_{i}}$ is the mean value and σ is the standard deviation of the data window. The result of the skew can be positive or negative.
  (A7) $$Skewness\left(x_{i}\right)=\frac{1}{L}\sum_{k=0}^{L-1}{\left(\frac{x_{i,k}-\bar{x_{i}}}{\sigma }\right)}^{3}$$

The next features are known as the Hjorth parameters: a set of three descriptors, originally developed to characterize EEG signals, which have been successfully shown to work for the recognition of EMG signals as well [28,55]. The Hjorth parameters are as follows:

8. Activity (Ac): the surface of the power spectrum in the frequency domain. It is calculated as the variance in the signal:
  (A8) $$Activity\left(x_{i}\right)=\frac{1}{L}\sum_{k=0}^{L-1}{\left(x_{i,k}-\bar{x_{i}}\right)}^{2}$$
9. Mobility (Mob): the representation of the mean frequency of the signal, where ${x’}_{i}$ is the first derivative of the signal with respect to time in the *i*-th window.
  (A9) $$Mobility\left(x_{i}\right)=\frac{Activity\left({x’}_{i}\right)}{Activity\left(x_{i}\right)}$$
10. Complexity (Cx): this represents the change in the frequency of the signal.
  (A10) $$Complexity\left(x_{i}\right)=\frac{Mobility\left({x’}_{i}\right)}{Mobility\left(x_{i}\right)}$$

## Appendix C. Activation, Training, and Performance Functions for NN in MATLAB

The *activation function* allows for the introduction of non-linearities into the prediction models, transforming the neural network values.

The selection of the activation function depends on the architecture and output of the neural network. In particular, the activation function of the output layer depends on the type of prediction problem. In classification problems between multiple classes, as is the case in the present work, the *softmax* activation function is recommended since the output is the probability of belonging to each class.

The activation function for the hidden layers must also be chosen. The Rectified Linear Unit function and enhanced versions are recommended for multi-layer perceptron.

The reader is referred to [51,56] and for an in-depth description of the concepts and explanation of these functions. The activation functions available in MATLAB are detailed in Table A1. The default activation functions in the MLP (*patternnet* function) are *tansig* for hidden layers and *softmax* for the output layer.

**Table A1:** Activation functions in MATLAB.

Activation Function	Description
*compet*	Competitive transfer function.
*elliotsig*	Elliot sigmoid transfer function.
*hardlim*	Positive hard limit transfer function.
*hardlims*	Symmetric hard limit transfer function.
*logsig*	Logarithmic sigmoid transfer function.
*netinv*	Inverse transfer function.
*poslin*	Positive linear transfer function.
*purelin*	Linear transfer function.
*radbas*	Radial basis transfer function.
*radbasn*	Radial basis normalized transfer function.
*satlin*	Positive saturating linear transfer function.
*satlins*	Symmetric saturating linear transfer function.
*softmax*	Soft max transfer function.
*tansig*	Symmetric sigmoid transfer function.
*tribas*	Triangular basis transfer function

The *training function* is the learning algorithm that allows a neural network to acquire a desired behavior by changing the weights of the interconnections. A list of available training functions in MATLAB for the MLP is detailed in Table A2 and have been gathered from [57].

**Table A2:** Training functions available in MATLAB.

Training Function	Acronym	Learning Algorithm
*trainlm*	LM	Levenberg–Marquardt
*trainbr*	BR	Bayesian Regularization
*trainbfg*	BFGS	BFGS Quasi-Newton
*trainrp*	RP	Resilient Backpropagation
*trainscg*	SCG	Scaled Conjugate Gradient
*traincgb*	CGB	Conjugate Gradient with Powell/Beale Restarts
*traincgf*	CGF	Fletcher–Powell Conjugate Gradient
*traincgp*	CGP	Polak–Ribiére Conjugate Gradient
*trainoss*	OSS	One Step Secant
*traingdx*	GDX	Variable Learning Rate Gradient Descent
*traingdm*	GDM	Gradient Descent with Momentum
*traingd*	GD	Gradient Descent backpropagation

The *performance function* measures the performance of the neural network while training. A list of available performance functions in MATLAB [58] is provided in Table A3. The default function is *crossentropy*.

**Table A3:** Performance functions in MATLAB.

Performance Function	Description
*mae*	Mean Absolute Error
*mse*	Mean Squared Normalized Error
*sae*	Sum Absolute Error
*sse*	Sum Square Error
*crossentropy*	Cross-entropy Calculation
*msesparse*	*MSE* with L2 weight and dispersion regulators
